# Development and validation of analytical methods for selective determination of albuterol and budesonide in Airsupra inhalation aerosol using spectrophotometry

**DOI:** 10.1038/s41598-023-42766-y

**Published:** 2023-10-03

**Authors:** Atiah H. Almalki, Sherif Ramzy, Ahmed A. Almrasy

**Affiliations:** 1 Department of Pharmaceutical Chemistry, College of Pharmacy, Taif University, P.O. Box 11099, 21944 Taif, Saudi Arabia; 2 Addiction and Neuroscience Research Unit, Health Science Campus, Taif University, P.O. Box 11099, 21944 Taif, Saudi Arabia; 3 Pharmaceutical Analytical Chemistry Department, Faculty of Pharmacy, Al-Azhar University, Cairo, 11751 Egypt

**Keywords:** Chemistry, Analytical chemistry

## Abstract

Airsupra inhalation aerosol is a recently approved FDA medication that combines albuterol and budesonide for treating or preventing bronchoconstriction and lowering the risk of relapses in asthma patients who are 18 years of age and older. To selectively determine albuterol and budesonide in both pure and pharmaceutical dosage forms, two analytical methods were developed: the zero-order absorption method and the dual-wavelength method. Even though the two drugs absorption spectra overlapped, the distinctive peak of budesonide at the zero absorbance point of albuterol, 245 nm, allowed for direct detection of budesonide in the combination using the zero-order absorption method. The mathematical dual-wavelength method, on the other hand, allowed for the measurement of both albuterol and budesonide by choosing two wavelengths for each drug in such a way that the absorbance difference for the second drug was zero. Budesonide exhibited comparable absorbance values at wavelengths 227 and 261.40 nm; hence, these two wavelengths were utilized to identify albuterol; similarly, 221.40 and 231.20 nm were chosen to determine budesonide in their binary mixes. The methods were validated according to the ICH guideline for validation of analytical procedures Q2(R1) and demonstrated excellent linearity, sensitivity, accuracy, precision, and selectivity for determining both drugs in synthetic mixed solutions and pharmaceutical formulations. The availability of these analytical methods would be valuable for the pharmaceutical industry and regulatory authorities for quality control and assessment of pharmaceutical formulations containing albuterol and budesonide.

## Introduction

Spectroscopy is a commonly analytical technique that allows the study of the interaction of many matters with electromagnetic radiation^1^. Through analyzing the spectral data produced by a sample, information about its chemical structure, and physical properties can be obtained. In the context of pharmaceutical analysis, spectroscopic methods are widely used to determine the purity, identity, and quantification of drugs in various types of samples, including pharmaceutical formulations, biological fluids, and environmental samples^2–9^.

**Figure 1:**
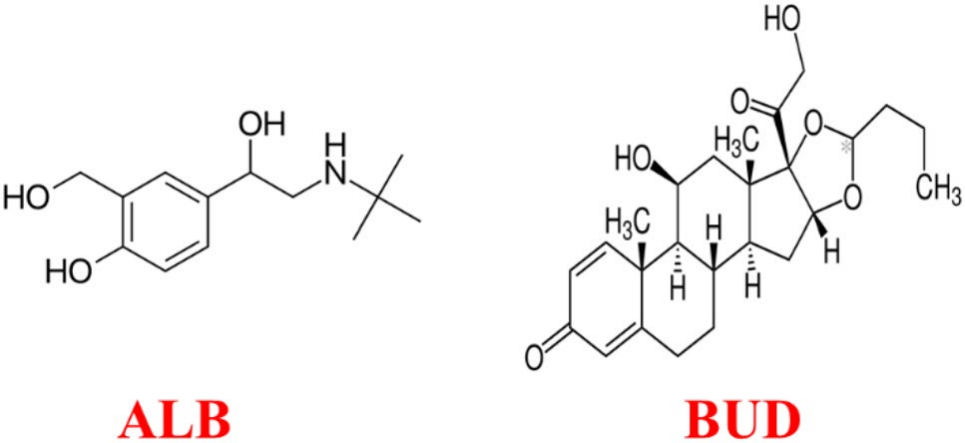
Chemical structures of ALB and BUD.

Airsupra inhalation aerosol is a newly approved FDA medication for treating or preventing bronchoconstriction and lowering the risk of relapses in asthma patients who are 18 years of age and older^10^. Airsupra combines two medicines, albuterol (ALB) and budesonide (BUD), in one oral inhaler, delivered into the airways as a propelled spray (Fig. 1). ALB is a rapid-acting beta2-adrenergic agonist medicine that works by relaxing the airway muscles, making it easier to breathe. BUD is an inhaled corticosteroid medicine that reduces inflammation in the airways, thereby preventing future asthma attacks. By combining these two medications in one inhaler, Airsupra offers an effective treatment for patients with asthma who need quick relief of their symptoms^11–13^.

Although ALB and BUD have been reported to be determined using the high-performance liquid chromatography technique in compounded nebulizer solutions including ipratropium bromide^14^, no spectroscopic methods for simultaneous quantification of ALB and BUD in a combination have been documented yet. As an alternative to HPLC, UV–visible spectrophotometry is the simplest and least expensive equipment available in all quality control labs for determining pharmaceutical compounds. It overcomes the drawbacks of HPLC, which generates more toxic waste and consumes more energy, hazardous solvents, and time.

The primary objective of this study is to develop analytical methods for the selective determination of ALB and BUD in a pharmaceutical formulation using UV–visible spectrophotometry. Despite the widespread use of these two medications in a combined oral inhaler, no spectroscopic methods have been documented for their selective quantification. This research gap necessitates the development of simple analytical techniques that can accurately quantify ALB and BUD. Addressing this gap is significant as it offers a cost-effective and environmentally friendly alternative to HPLC methods, which are commonly used for drug analysis but have associated drawbacks. By bridging this research gap, the developed methods can provide valuable tools for quality control and assessment of the Airsupra inhalation aerosol, benefiting both pharmaceutical research and regulatory bodies.

In this study, the authors investigated the spectral characteristics of two drugs, ALB, and BUD, to develop analytical methods for their selective determination in a pharmaceutical formulation. The spectral characteristics of ALB and BUD were determined by measuring their UV absorption spectra, the results showed that the absorption spectra of ALB and BUD overlapped. Although the overlapping, the defined characteristic peak of BUD at 245 nm, where there was no absorbance overlap with ALB, enabled direct determination of BUD in the mixture using the zero-order absorption method^15–19^ with no interference from ALB. Unfortunately, because ALB did not have absorption peaks at zero absorbance points of BUD, the zero-order method did not allow for the simultaneous identification of the two drugs. The dual-wavelength method^20,21^, on the other hand, allowed for the measurement of both ALB and BUD in their combination. The dual-wavelength method depends on selecting two wavelengths for each drug so that the absorbance difference for the second drug is zero. BUD had similar absorbance values at wavelengths 227 and 261.40 nm, hence these two wavelengths were used to identify ALB; similarly, 221.40 and 231.20 nm were selected to identify BUD in their binary mixtures. The methods used were straightforward, requiring no complicated mathematical manipulation. The methods were validated according to the ICH guideline for validation of analytical procedures Q2(R1)^22^, and showed excellent linearity, sensitivity, accuracy, precision, and selectivity for the determination of ALB and BUD in synthetic mixed solutions and pharmaceutical formulations.

## Experimental

### Materials and solvent

The reference standard powders of ALB and BUD, as well as the Airsupra metered dose inhaler (ALB 90 µg and BUD 80 µg), were provided by the National Organization for Drug Control and Research (Egypt). Ethanol for the analysis was obtained from El-Nasr Company (Egypt).

### Apparatus

All measurements were taken with a Shimadzu UV–Visible 1800 Spectrophotometer (Japan) and UV-Probe software version 2.43.

### Preparation of standard solutions

The standard stock solutions of ALB and BUD, each at a concentration of 100 µg/mL, were prepared separately in ethanol.

### Preparation of procedure samples

#### Preparation of the calibration standards

ALB and BUD calibration standards were prepared separately at eight concentration levels. Aliquots of 30, 50, 100, 150, 200, 300, 400, and 500 µg ALB and 20, 50, 100, 150, 200, 250, 300, and 400 µg BUD were transferred from standard stock solutions into two separate sets of 10-mL volumetric flasks. The flasks were then diluted with ethanol to yield calibration standards of 3, 5, 10, 15, 20, 30, 40, and 50 µg/mL for ALB and 2, 5, 10, 15, 20, 25, 30, and 40 µg/mL for BUD.

#### Preparation of the synthetic mixed solutions

Five synthetic mixed solutions of ALB and BUD were made in the same pharmaceutical formulation concentration ratio of 1.25:1. Aliquots of 45, 90, 180, 225, and 270 µg ALB were mixed in sequence with aliquots of 40, 80, 160, 200, and 240 µg BUD in a series of 10-mL volumetric flasks. After dilution with ethanol, the flasks yielded final concentrations of ALB and BUD (µg/mL) of 4.5:4, 9:8, 18:16, 22.5:20, and 27:24, respectively.

#### Preparation of the pharmaceutical samples

The pressurized canister was removed from the actuator and placed upright in a plastic bag. After cooling to − 20 °C for 30 min, the canister's shoulder was carefully pierced to form a small hole that allowed the propellants to evaporate. The valve and the top of the canister were then removed and washed with ethanol. The canister was then filled with 10 mL of ethanol and sonicated to dissolve at room temperature. The contents of the canister were transferred to a volumetric flask of 100 mL. Repeat the procedure above with 2 × 10 mL of ethanol and sonicate until dissolved. The canister was rinsed with diluent once more. The volumetric flask was then filled to capacity with ethanol. Further dilutions were performed in order to prepare five samples containing final concentrations of ALB and BUD (µg/mL) of 4.5:4, 9:8, 18:16, 22.5:20, and 27:24, respectively.

#### Preparation of the standard addition samples

Three pharmaceutical aliquots, each containing 90 µg ALB and 80 µg BUD, were spiked separately in a set of 10-mL volumetric flasks with aliquots of pure ALB (45, 90, and 180 µg) and pure BUD (40, 80, and 160 µg). The flasks were then thoroughly mixed and filled with ethanol to capacity.

### Method development and validation

#### Linearity and calibration graphs

The absorption spectra of the calibration standards of the studied drugs were measured against ethanol as a blank in the wavelength range from 200 to 400 nm.

For the zero-order method, the absorbance values at 245 nm were directly correlated to BUD with no interference from ALB, which has no absorbance. The calibration curve was constructed by plotting the absorbance values of BUD at 245 nm against the corresponding drug concentrations ranging from 2 to 40 µg/mL. The coefficient of determination, y-intercept and slope of the regression line were then derived.

For the dual wavelength method, the difference in absorbance values at 227 and 261.40 nm for ALB (difference is zero for BUD) and at 221.40 and 231.20 nm for BUD (difference is zero for ALB) were calculated. The calibration curve for ALB was constructed by plotting the difference in absorbance values at 227 and 261.40 nm against the corresponding drug concentrations ranging from 3 to 50 µg/mL. Similarly, the calibration curve for BUD was constructed by plotting the difference in absorbance values at 221.40 and 231.20 nm against the corresponding drug concentrations ranging from 2–40 µg/mL. The coefficient of determination, y-intercept and slope of the regression line were then derived.

#### Detection limit and quantitation limit

The detection limit (DL) and quantitation limit (QL) of the applied methods were estimated based on the residual standard deviation (σ) and a slope (S). The detection limit was given as DL = 3.3σ/S and the quantitation limit was given as QL = 10σ/S^22^.

#### Accuracy

The accuracy of the applied methods was calculated as the mean percent recovery of three replicate determinations of synthetic mixed solutions using the procedure described in the linearity and calibration graphs.

#### Precision

The precision of the applied methods was calculated using the procedure described in the linearity and calibration graphs as the relative standard deviation of three replicate determinations of the synthetic mixed solutions on the same day for repeatability precision and three days for intermediate precision.

#### Selectivity

The ability of the applied methods for selective quantification of the studied drugs with no interference was investigated by analyzing synthetic mixed solutions, pharmaceutical samples, and standard addition samples using the procedure described in the linearity and calibration graphs.

## Results and discussion

### Spectral characteristics

**Figure 2:**
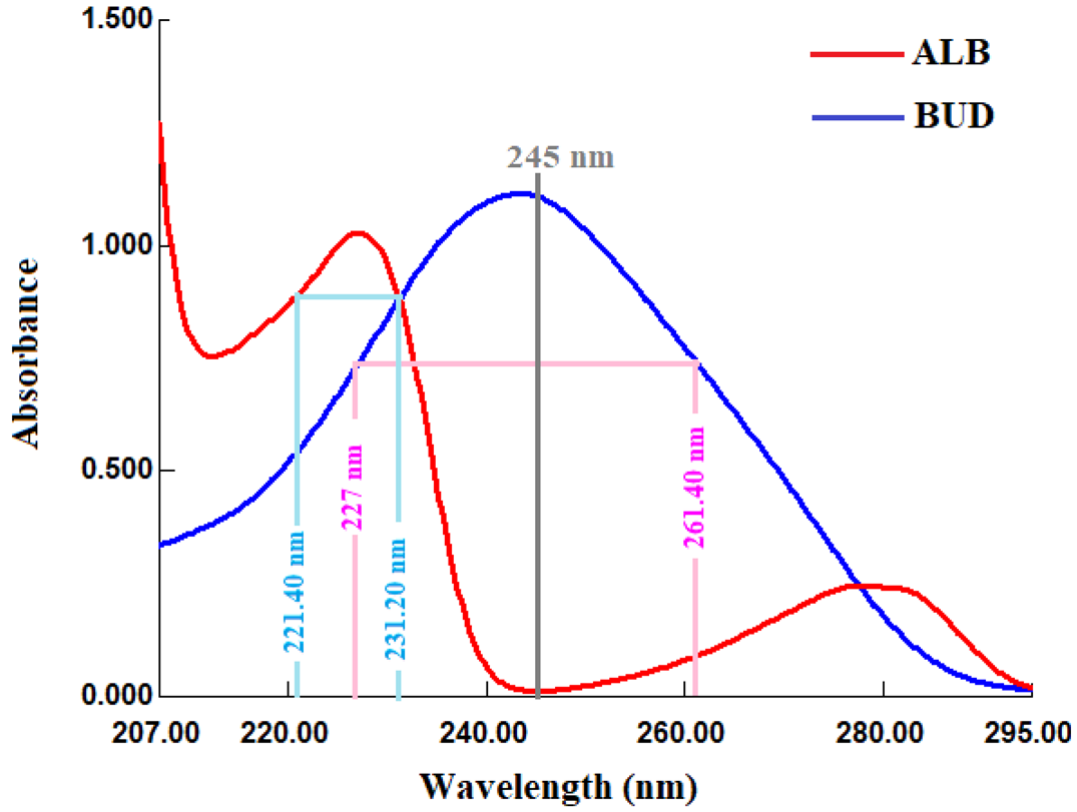
Absorption spectra of ALB (40 μg/mL) and BUD (40 μg/mL).

The ALB and BUD UV absorption spectra were totally overlapped, however the BUD spectra exhibit a distinctive peak at 245 nm, where there is no absorbance response for ALB (Fig. 2). As a result, BUD could be detected directly in the combination at 245 nm using the zero-order absorption method with no interference from ALB, but ALB could not be determined directly in the mixture. The determination of both ALB and BUD in the combination was made possible using the mathematical dual-wavelength method.

#### Zero-order absorption method

The zero-order absorption method is based on determining the absorbance values of the drug of interest at the point of zero absorbance of other substances in the mixture. BUD has an absorption peak at 245 nm, which is the zero-absorbance point of ALB. Therefore, the absorbance values of at 245 nm were directly proportional the concentrations of BUD without any interference from ALB (Fig. 2). In contrast to the absorption correction method, which requires the calculation of correction factors, which may affect the accuracy of the results, the zero-order absorption method is a simple method that processes the zero-absorption spectra without any mathematical manipulations and produces highly accurate results.

#### Dual-wavelength method

The dual-wavelength method depends on selecting two wavelengths for each drug so that the absorbance difference for the second drug is zero. As a result, for ALB determination, the difference in absorbance values between several pairs of wavelengths (the difference is zero for BUD) was investigated in terms of ALB linearity. Good linearity of ALB was achieved with a wavelength pair of 227 and 261.40 nm. The difference in absorbance values at 227 and 261.40 nm was correlated to ALB concentrations without any influence from BUD, as shown in Fig. 2. Similarly, for BUD determination, the difference in absorbance values between several pairs of wavelengths (the difference is zero for ALB) was investigated in terms of BUD linearity. Good linearity of BUD was achieved with a wavelength pair of 221.40 and 231.20 nm. The difference in absorbance values at 221.40 and 231.20 nm was correlated to BUD concentrations without any influence from ALB, as shown in Fig. 2.

### Methods validation

The applied methods were validated according to ICH guideline for validation of analytical procedures Q2(R1)^22–26^. Validation characteristics that were evaluated included linearity and range, detection limit (DL) and quantitation limit (QL), accuracy, precision, and selectivity.

#### Linearity and range

Linearity was evaluated using eight calibration standard concentration levels ranging from 3–50 µg/mL for ALB and 2–40 µg/mL for BUD. In the zero-order absorption method, a linear relationship for BUD was achieved by graphing BUD absorbance signals at 245 nm against the respective concentrations with a correlation coefficient of 0.9999. In the dual wavelength method, on the other hand, by graphing the difference in absorbance values at 227 and 261.40 nm for ALB and at 221.40 and 231.20 nm for BUD against the corresponding drug concentrations, a linear relationship for ALB and BUD was achieved with a correlation coefficient of 0.9998 and 0.9999, respectively. The linearity data are presented in Table 1.

**Table 1:** Regression and validation data for quantitative analysis of ALB and BUD by the proposed methods.

Parameters	Zero-order method	Dual wavelength method	
	BUD	ALB	BUD
Wavelength (nm)	245	227 and 261.40	221.40 and 231.20
Linearity range (µg/mL)	2–40	3–50	2–40
Slope	0.0273	0.0238	0.0080
Intercept	0.0153	− 0.0101	0.0048
Coefficient of determination (r^2^)	0.9999	0.9998	0.9999
DL* (µg/mL)	0.317	0.696	0.476
QL** (µg/mL)	0.962	2.109	1.444

*DL = 3.3σ/S.

**QL = 10σ/S.

#### Detection limit (DL) and quantitation limit (QL)

The detection limit (DL = 3.3σ/S) and quantitation limit (QL = 10σ/S) of the applied methods were calculated using the residual standard deviation (σ), and a slope (S). Table 1 displays the computed DL and QL values. The low DL and QL results showed the sensitivity of the procedures used.

#### Accuracy

The accuracy of the applied methods was calculated as the mean percent recovery of three replicate determinations of the five synthetic mixed solutions (n = 15). The methods demonstrated excellent accuracy results as displayed in Table 2.

**Table 2:** Accuracy and repeatability precision of the proposed methods using synthetic mixed solutions (n = 15).

#	Added (µg/mL)		Percent recovery		
	ALB	BUD	Zero-order method	Dual wavelength method	
			BUD	ALB	BUD
Run 1	4.5	4	99.54	98.13	99.17
	9	8	99.68	99.49	98.06
	18	16	98.37	99.00	98.06
	22.5	20	99.85	99.74	99.00
	27	24	98.86	99.92	99.17
Run 2	4.5	4	98.63	99.07	98.61
	9	8	99.68	98.09	99.58
	18	16	99.29	98.53	98.75
	22.5	20	99.95	99.55	99.56
	27	24	99.92	99.92	98.70
Run 3	4.5	4	99.54	97.20	99.17
	9	8	98.31	99.02	99.03
	18	16	99.98	99.93	99.31
	22.5	20	98.30	98.99	98.44
	27	24	99.62	99.61	99.63
Accuracy (Mean)			99.30	99.08	98.95
Repeatability precision (RSD)			0.636	0.809	0.511

#### Precision

The precision of the applied methods was calculated as the relative standard deviation of three replicate determinations of the five synthetic mixed solutions (n = 15) on the same day for repeatability precision (Table 2) and three days for intermediate precision (Table 3). The methods demonstrated a high level of precision, with an RSD of less than 2%.

**Table 3:** Intermediate precision of the proposed methods using synthetic mixed solutions (n = 15).

#	Added (µg/mL)		Percent recovery		
	ALB	BUD	Zero-order method	Dual wavelength method	
			BUD	ALB	BUD
Day 1	4.5	4	99.54	98.13	99.17
	9	8	99.68	99.49	98.06
	18	16	98.37	99.00	98.06
	22.5	20	99.85	99.74	99.00
	27	24	98.86	99.92	99.17
Day 2	4.5	4	98.63	98.13	99.17
	9	8	98.76	99.95	99.86
	18	16	99.52	99.70	99.44
	22.5	20	99.76	99.18	98.44
	27	24	99.16	98.68	99.17
Day 3	4.5	4	98.63	97.20	98.06
	9	8	99.22	98.55	99.31
	18	16	98.37	99.23	99.93
	22.5	20	99.76	98.06	99.56
	27	24	99.16	99.61	99.12
Mean			99.15	98.97	99.03
Intermediate precision (RSD)			0.526	0.828	0.621

#### Selectivity

The methods used demonstrated high selectivity with good accuracy and precision for determining ALB and BUD in their synthetic mixed solutions (Table 2) with no interference from each other, as well as in pharmaceutical formulation (Table 4) with no interference from pharmaceutical excipients. Furthermore, the results of the standard addition technique shown in Table 5 confirm the selectivity of the methods used.

**Table 4:** Application of the proposed method for the determination of ALB and BUD in pharmaceutical dosage form.

Taken (µg/mL)		Percent recovery		
		Zero-order method	Dual wavelength method	
ALB	BUD	BUD	ALB	BUD
4.5	4	98.63	98.13	98.61
9	8	99.68	98.55	99.17
18	16	99.29	99.93	99.44
22.5	20	98.85	99.37	99.56
27	24	99.77	99.77	99.63
Mean ± RSD		99.24 ± 0.506	99.15 ± 0.786	99.28 ± 0.417

**Table 5:** Recovery study of ALB and BUD by standard addition technique using the proposed methods.

Amount of drug (µg/mL)		Amount of pure added (µg/mL)		Percent recovery		
				Zero-order method	Dual wavelength method	
ALB	BUD	ALB	BUD	BUD	ALB	BUD
9	8	4.5	4	98.86	99.65	99.21
		9	8	99.34	98.84	98.22
		18	16	99.81	99.14	98.41
Mean ± RSD				99.33 ± 0.477	99.21 ± 0.410	98.62 ± 0.535

### Application of the methods

The proposed methods were successful in quantifying ALB and BUD in their synthetic mixed solutions (Table 2) with no interference from each other, and pharmaceutical formulation (Table 4) with no interference from excipients, as confirmed by the results of the standard addition technique, Table 5.

## Conclusion

Two analytical methods, the zero-order absorption and the dual-wavelength, have been developed to selectively determine albuterol and budesonide in their pure form or in pharmaceutical formulations. The described methods demonstrated high levels of accuracy, precision, sensitivity, and selectivity and were validated according to established industry guidelines. These analytical methods could be of significant value to regulatory bodies and the pharmaceutical industry for quality control and assessment purposes related to Airsupra® inhalation aerosol.

## Data Availability

The datasets used and/or analyzed during the current study available from the corresponding author on reasonable request.
